# Shiga Toxin (Stx)-Binding Glycosphingolipids of Primary Human Renal Cortical Epithelial Cells (pHRCEpiCs) and Stx-Mediated Cytotoxicity

**DOI:** 10.3390/toxins13020139

**Published:** 2021-02-12

**Authors:** Johanna Detzner, Elisabeth Krojnewski, Gottfried Pohlentz, Daniel Steil, Hans-Ulrich Humpf, Alexander Mellmann, Helge Karch, Johannes Müthing

**Affiliations:** 1 Institute of Hygiene, University of Münster, D-48149 Münster, Germany; johanna.detzner@ukmuenster.de (J.D.); lilly.kroj@gmail.com (E.K.); pohlentz@uni-muenster.de (G.P.); daniel.steil87@gmail.com (D.S.); Alexander.Mellmann@ukmuenster.de (A.M.); hkarch@uni-muenster.de (H.K.); 2 Institute of Food Chemistry, University of Münster, D-48149 Münster, Germany; humpf@uni-muenster.de

**Keywords:** pHRCEpiCs, EHEC, kidney cortical epithelial cells, glycolipids, receptor, Shiga toxin, Stx1a, Stx2a, STEC

## Abstract

Human kidney epithelial cells are supposed to be directly involved in the pathogenesis of the hemolytic–uremic syndrome (HUS) caused by Shiga toxin (Stx)-producing enterohemorrhagic *Escherichia coli* (EHEC). The characterization of the major and minor Stx-binding glycosphingolipids (GSLs) globotriaosylceramide (Gb3Cer) and globotetraosylceramide (Gb4Cer), respectively, of primary human renal cortical epithelial cells (pHRCEpiCs) revealed GSLs with Cer (d18:1, C16:0), Cer (d18:1, C22:0), and Cer (d18:1, C24:1/C24:0) as the dominant lipoforms. Using detergent-resistant membranes (DRMs) and non-DRMs, Gb3Cer and Gb4Cer prevailed in the DRM fractions, suggesting their association with microdomains in the liquid-ordered membrane phase. A preference of Gb3Cer and Gb4Cer endowed with C24:0 fatty acid accompanied by minor monounsaturated C24:1-harboring counterparts was observed in DRMs, whereas the C24:1 fatty acid increased in relation to the saturated equivalents in non-DRMs. A shift of the dominant phospholipid phosphatidylcholine with saturated fatty acids in the DRM to unsaturated species in the non-DRM fractions correlated with the GSL distribution. Cytotoxicity assays gave a moderate susceptibility of pHRCEpiCs to the Stx1a and Stx2a subtypes when compared to highly sensitive Vero-B4 cells. The results indicate that presence of Stx-binding GSLs per se and preferred occurrence in microdomains do not necessarily lead to a high cellular susceptibility towards Stx.

## 1. Introduction

Shiga toxins (Stxs) are potent bacterial exotoxins and the principal virulence factors of pathogenic enterohemorrhagic *Escherichia coli* (EHEC), a subset of Stx-producing *E. coli* (STEC) [1]. They are responsible for major clinical manifestations of STEC infections, including bloody diarrhea and life-threatening extraintestinal complications such as the hemolytic–uremic syndrome (HUS) and neurological disturbances as the most serious consequences of these infections [2,3,4,5]. An update on global outbreaks of STEC and its potential reservoirs has recently been published [6]. Stxs belong to the group of AB_5_ enterotoxins [7]. The B-pentamer of the Stx1a and Stx2a subtypes preferentially binds to the glycosphingolipid (GSL) globotriaosylceramide (Gb3Cer) and to less extent to globotetraosylceramide (Gb4Cer) primarily found in human endothelial cells of various vascular beds [8,9,10,11,12,13]. Subsequent endocytosis of the toxin–GSL complex and retrograde transport occurs by multifarious endocytic routes directing the toxin via early endosomes through the Golgi apparatus to the endoplasmic reticulum [14,15,16,17]. Upon translocation into the cytosol, the *N*-glycosidase activity of the A1 moiety of the A subunit releases a single adenine residue from the 28S rRNA and brings protein synthesis at the ribosomes to a halt [7,18]. Moreover, Stx is capable of efficiently depurinating DNA in the cell nucleus [19,20], indicating the existence of at least more than one intracellular target structure. In addition, and probably even more important, Stxs are multi-functional proteins and capable of modulating a plethora of essential cellular functions at the molecular level such as the induction of signaling cascades that lead to apoptosis [1,21,22]. Compounds and conditions found to protect cells against Stxs have been reviewed [23], and, for example, glycovesicles spiked with neoglycolipids carrying Gb3 or (α1-4Gal)_n_ oligosaccharides have been recently reported to have an effect as inhibitors of Stx-mediated cellular injury [24,25].

Outer membrane vesicles (OMVs) serve as cargo vehicles for virulence factors, including Stx2a released by EHEC O157:H7 and O104:H4 clinical isolates [26,27]. The OMVs bind to and are internalized by human intestinal epithelial cells and deliver the OMV-associated Stx2a intracellularly, representing a novel way to deliver pathogenic cargoes and injure host cells [26]. Importantly, the intrahost milieu was found to modulate the OMV production, OMV-associated Stx2a, and cytotoxicity in EHEC O157:H7 and O104:H4 [28]. Once delivered into the colon, Stx gains access to the blood stream, where granulocytes are discussed over a period of time as transporters of Stx through the circulation of the toxin to the endothelium [29,30,31,32]. On the other side, blood cell-derived microvesicles, shed during HUS by Stx-stimulated blood cells and loaded with Stx, were discovered as a novel virulence mechanism [33]. Stx-containing microvesicles are taken up by glomerular endothelial cells in vitro and undergo endocytosis, leading to inhibition of protein synthesis and cell death [33,34]. Importantly, Stx-bearing microvesicles exert a cytotoxic effect on recipient cells only when the cells do express the toxin’s receptor GSL Gb3Cer [35]. Moreover, an association of vesicular Stx2 in blood to the development of HUS, i.e., in the transition period from hemorrhagic colitis to HUS, was found in STEC-infected children [36]. Thus, vesicles derived from host cells represent a further new strategy whereby Stx is transferred by microvesicles in which the toxin may evade the host immune system.

It is generally accepted that Stxs target mainly microvascular endothelial cells of the renal glomeruli and the brain in humans [1,13,18,37,38], although evidence has been provided in an ex vivo model using human hematopoietic stem/progenitor cells that erythropoiesis, which takes place in the bone marrow, may be affected by Stx [39,40]. Thus, Stx-mediated toxicity towards erythroblasts during the course of erythropoiesis might contribute to anemia observed during manifestation of STEC-HUS. Moreover, renal proximal tubular epithelial cells, mesangial cells, and podocytes, as well as intestinal epithelial cells and monocytes/macrophages, are susceptible to Stx-mediated injury in humans [41,42,43,44,45,46]. However, human kidney and brain microvascular endothelial cells are widely used to explore the impact of Stx-mediated cellular dysfunction because of the association of endothelial injury in the kidney with HUS and due to neurological impairment of the brain being the most frequent cause of acute mortality in patients with STEC-HUS [38,47,48,49,50].

Stx-mediated kidney injury and organ failure may also result to a great extent from direct effects of the toxin not only on endothelial but on renal epithelial cells [51,52,53]. A number of studies have shown that the renal cell lines HK-2 [54,55,56,57,58,59] and ACHN [60,61,62,63,64] derived from human tubule epithelium are sensitive to Stx, suggesting that injuries to this epithelium are involved in the development of acute renal failure in HUS. HK-2 and ACHN cells harbor globotriaosylceramide (Gb3Cer) [58,60,62], and the various lipoforms of Stx-binding Gb3Cer of the ACHN cell line have been recently identified [65]. Furthermore, Stx-mediated cytopathic action has also been shown for primary human renal glomerular and tubular epithelial cells [66,67,68,69,70,71,72], suggesting contribution of renal epithelial cells in Stx-mediated kidney failure as previously shown in a mouse model [73,74]. To warrant functionally intact primary cells and to avoid spontaneous decay of their differential phenotype, it is mandatory to restrict the total number of cell passages (commonly ≤ 8 passages). For this reason, the ex vivo production of sufficient cell quantities for the isolation of GSLs and for membrane preparations is seriously limited. However, the principal advantages over tumor-derived or immortalized cell lines lies in the more faithful mimicry of the differentiated in vivo pheno- and geno-type of primary cells providing the closest in vitro model to human organs [75,76,77,78,79]. However, primary cells exhibit stringent nutritional demands and need dedicated cell culture media, especially for cultivation under serum-free conditions requiring certain growth-promoting agents, such as growth factors, lipids, and/or hormones to compensate for serum deprivation [80,81,82,83].

Based on the pioneering work of Brown and Rose, the recovery of glycosylphosphatidylinositol (GPI)-anchored proteins together with GSLs in detergent-resistant vesicles prepared from lysates of cultured kidney epithelial cells suggested sorting of certain membrane proteins to the apical surface after intracellular association with GSLs [84]. The insolubility of lipids in detergents such as Triton X-100 represents a practicable method for exploring the structure of biological membranes and has become an invaluable tool for characterizing the composition of cellular membrane microdomains, also known as *lipid rafts* [85,86,87]. Enrichment in detergent-resistant membranes (DRMs) provides a good indication that a membrane constituent is “raftophilic” and may be present in *lipid rafts* before extraction [88]. Although DRMs are artifactual in nature and not the same as native *lipid rafts*, probing of DRMs is often beneficial for analyzing protein function, implicating *lipid rafts* in a myriad of cellular processes [89]. It is well accepted that the lipid phase of the *rafts* complies with the liquid-ordered membrane phase and that such domains are more tightly packed than the liquid-disordered membrane phase. This clustering, most likely due to hydrophobic interactions involving the canonical *lipid raft* markers cholesterol, sphingomyelin, and GSLs, explains the resistance of *rafts* towards detergent solubilization, while the liquid-disordered membrane phase corresponds to detergent-soluble membranes [85,89,90]. *Lipid rafts* represent a concept of membrane subcompartmentalization forming platforms that function in membrane signaling and trafficking [91]. Moreover, *lipid rafts* are targeted by a wide variety of pathogens to invade host cells as an infectious strategy and to escape from the immune system by hijacking microdomains in the plane of the cell membrane [92,93,94,95,96,97]. GSLs that reside in the exofacial leaflet of the plasma membrane microdomains and strongly associate with DRMs are excellent candidates as recognition structures for numerous bacterial toxins of the group of AB_5_ toxins, including Stx [18,98,99,100]. The pentameric B-subunit of Stx interacts with GSLs by unusual membrane-biological concepts, such as fluctuation force-driven scission, membrane-curvature generation, friction-driven scission, and retrograde sorting on early endosomes [15,101,102,103,104,105,106]. The use of DRMs has proven a successful approach for detecting lysosomal degradation and failure of retrograde transportation of the Stx B-subunit in monocyte-derived cells [107] as well as showing the association of Stx with DRMs and high Gb3Cer density within DRMs as essential requirements for a cytotoxic effect [108,109] and the occurrence of functionally different pools of Stx-binding Gb3Cer in HeLa cells [110]. Importantly, detergent-resistant Gb3Cer in human glomeruli and detergent-sensitive Gb3Cer in renal tubules indicated that different ceramide lipoforms of Gb3Cer may play a functional role in glomerular endothelial and tubular epithelial cells and may define restricted Stx pathology in HUS to glomeruli [13,111,112]. The fine structures of Stx-binding GSLs of primary human renal epithelial cells and their distribution in the cell membrane as well as their vicinal membrane lipids have not been analyzed yet. Here, we report on the comprehensive structural analysis of the various lipoforms of Stx-binding GSLs isolated from primary human renal cortical epithelial cells (pHRCEpiCs) and their distribution to DRMs and non-DRMs used as counterparts for the liquid-ordered and the liquid-disordered membrane phase, respectively. In addition, we determined the unexpected low susceptibility of pHRCEpiCs towards the clinically highly relevant Stx1a and Stx2a subtypes.

## 2. Results

This study starts with the identification and structural characterization of Stx-binding GSLs of the globo-series isolated from two replicates of primary human renal cortical epithelial cells (pHRCEpiCs). To this end, pHRCEpiCs were propagated until passage 8 and then submitted to lipid isolation and GSL as well as phospholipid analysis. Although these primary kidney epithelial cells can be cultured beyond passage 10, they began to show microscopic signs of dedifferentiation and senescence beyond passage 10 (see Figure S1 in the Supplementary Materials). The two principle subtypes, Stx1a and Stx2a (for standardized nomenclature of Stx subtypes, refer to Scheutz and collaborators [113]), were employed as affinity-purified samples in thin-layer chromatography (TLC) overlay assays for the detection of Stx-binding GSLs. The identified Stx receptor GSLs were further structurally characterized by TLC overlay immunodetection using anti-Gb3Cer and anti-Gb4Cer antibodies combined with electrospray ionization mass spectrometry (ESI-MS). After analysis of total GSLs of pHRCEpiCs, their association with the liquid-ordered and liquid-disordered membranes was probed using DRM and non-DRM fractions, respectively, obtained from sucrose gradient ultracentrifugation. The ambient phospholipids were analyzed in detail by MS^1^ and MS^2^ analysis, the latter performed by collision-induced dissociation (CID) experiments. The study ends with determining the susceptibility of the pHRCEpiCs towards Stx1a and Stx2a in comparison to the Vero-B4 reference cell line.

### 2.1. Identification of Stx1a- and Stx2a-Binding Globo-Series Glycosphingolipids of pHRCEpiCs

**Figure 1:**
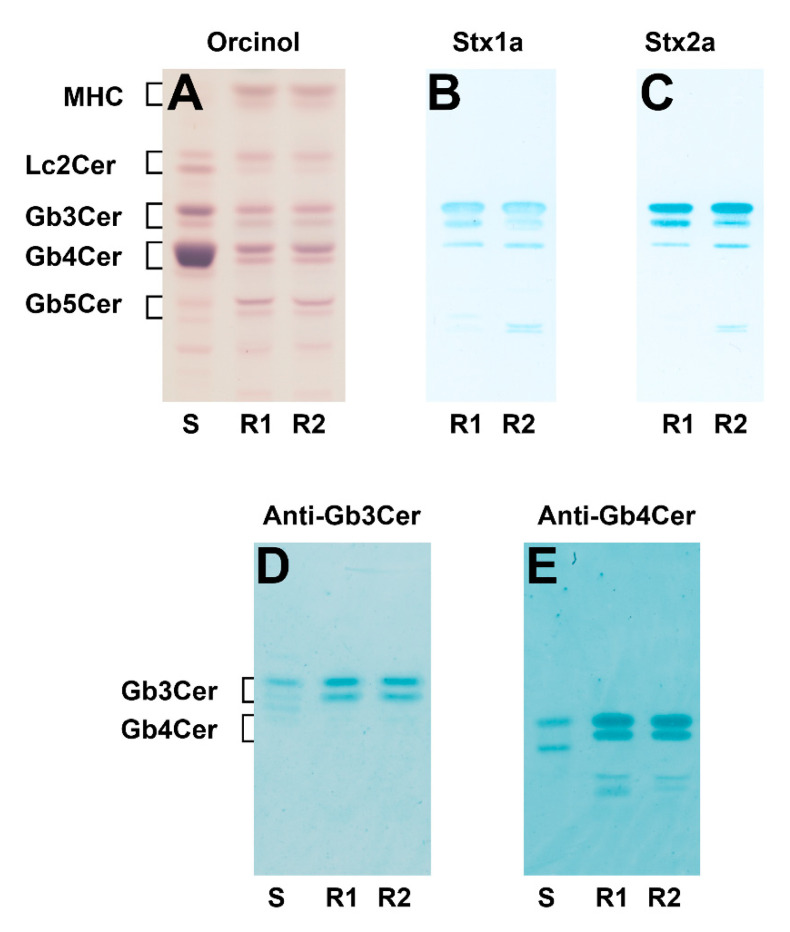
Orcinol stain (**A**) and Shiga toxin (Stx)1a (**B**), Stx2a (**C**), anti-globotriaosylceramide (Gb3Cer) (**D**), and anti-globotetraosylceramide (Gb4Cer) (**E**) overlay assay of thin-layer chromatography (TLC)-separated neutral glycosphingolipids (GSLs) from primary human renal cortical epithelial cells (pHRCEpiCs). The applied GSL amounts correspond to 2 × 10^6^ cells for the orcinol stain (**A**), 4 × 10^5^ cells for the Stx1a (**B**) and Stx2a (**C**), as well as 2 × 10^5^ cells for the anti-Gb3Cer (**D**) and the anti-Gb4Cer (**E**) TLC overlay assay, respectively. S, standard: 20 µg for the orcinol stain (**A**), 2 µg for the anti-Gb3Cer, (**D**) and 0.2 µg for the anti-Gb4Cer (**E**) immunostain of neutral GSLs from human erythrocytes; R1, replicate 1; R2, replicate 2; MHC, monohexosylceramides.

Lipids were extracted from in vitro propagated pHRCEpiCs, followed by GSL isolation using anion-exchange chromatography. Neutral GSLs were analyzed by thin-layer chromatography (TLC) as portrayed in Figure 1. The orcinol stain of TLC-separated GSLs revealed, besides monohexosylceramides (MHC) and lactosylceramide (Lc2Cer), the apparent presence of Gb3Cer, Gb4Cer, and Gb5Cer in the GSL samples of both replicates (R1 and R2) of pHRCEpiCs, as shown in Figure 1A, when compared to standard GSLs. The TLC overlay assay using Stx1a and Stx2a indicates strong binding towards Gb3Cer and less intensive recognition of Gb4Cer, as displayed for the two replicates in Figure 1B and Figure 1C, respectively. The Stx-positive Gb3Cer double bands correspond to the orcinol-stained double bands regarding position and intensity, suggesting higher relative content of the upper compared to the lower band, whereby the upper band bears Gb3Cer lipoforms with long-chain (C22–C24) fatty acids and the lower one harbors Gb3Cer with short-chain (commonly C16) fatty acids. These Gb3Cer species correlate with those of Stx-binding Gb3Cer variants previously detected in primary human brain and kidney endothelial cells [12]. Both Stx1a and Stx2a bind relatively weakly to the upper predominant band of the Gb4Cer doublet of both replicates (R1 and R2) (Figure 1B,C), representing most likely Gb4Cer lipoforms with a long-chain fatty acid but not to the lower migrating Gb4Cer species. This underlines the preference of Stx1a and Stx2a to Gb3Cer over Gb4Cer as known from earlier binding studies [12]. The proof of the proposed Gb3 and Gb4 oligosaccharides is shown in Figure 1D and Figure 1E, using anti-Gb3Cer and anti-Gb4Cer antibodies, respectively. Thus, Gb3Cer as well as Gb4Cer exhibit identical double band pattern in the two replicates (R1 and R2). Interestingly, only the upper band of Gb4Cer is recognized by the two Stx subtypes (see Figure 1B,C) using the TLC overlay assay.

### 2.2. Structural Characterization of Sphingolipids from the Neutral GSL Fraction of pHRCEpiCs

**Figure 2:**
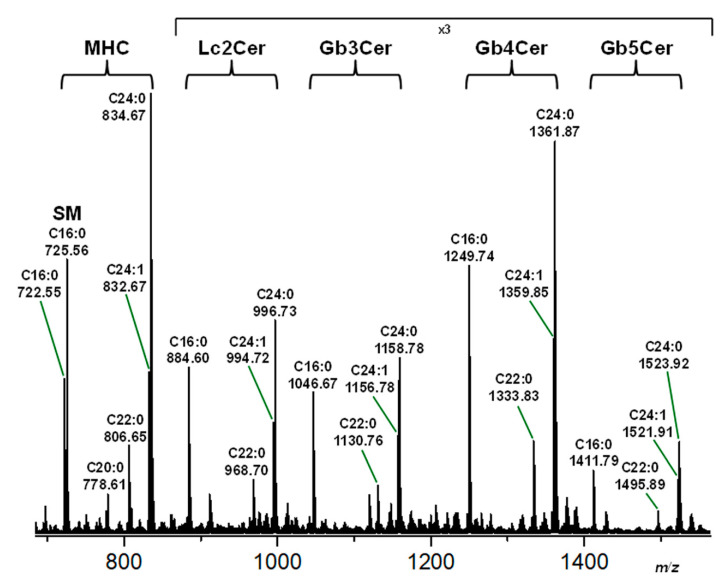
Overview MS^1^ spectrum of the neutral GSL preparation of pHRCEpiCs. The neutral GSL sample was obtained from replicate 1 of pHRCEpiCs (see orcinol stain of R1 in Figure 1). The identified mono-, di-, tri-, tetra-, and penta-hexosylceramides were assigned to monohexosylceramide (MHC), lactosylceramide (Lc2Cer), Gb3Cer, Gb4Cer, and Gb5Cer (see Stx1a and Stx2a as well as anti-Gb3Cer and anti-Gb4Cer TLC overlay assays of R1 in Figure 1). Sphingomyelin (SM) and GSLs were detected as monosodiated [M+Na]^+^ ions using the positive ion mode and are listed in Table 1. In-depth structural details are exemplified by MS^2^ spectra of Gb3Cer (d18:1, C16:0), Gb4Cer (d18:1, C22:0), and proposed Gb5Cer (d18:1, C16:0), which are given in Figure S2, Figure S3 and Figure S4, respectively, of the Supplementary Materials.

Initial verification of the structures of the sphingolipids is demonstrated by the positive ion MS^1^ spectrum obtained from the neutral GSL preparation of pHRCEpiCs (replicate 1) shown in Figure 2. All analytes arose as monsodiated [M+Na]^+^ ions and suggested considerable structural heterogeneity of expected GSLs in their ceramide moieties. The exact structures of proposed GSL species were elucidated by CID MS analysis and revealed MHC (Gal/Glcβ1Cer) and Lc2Cer (Galβ4Glcβ1Cer) as well as Stx- and antibody-detected Gb3Cer (Galα4Galβ4Glcβ1Cer) and Gb4Cer (GalNAcβ3Galα4Galβ4Glcβ1Cer) lipoforms harboring sphingosine (d18:1) as the sole amino alcohol in their ceramide moieties being linked to a C16:0, C22:0, or C24:0/C24:1 fatty acid as indicated in the spectrum and listed in Table 1. Minor Gb3Cer lipoforms were those with Cer (d18:1, C24:2) and Cer (d18:1, C26:1/C26:0) at *m*/*z* 1154.76 and 1184.80/1186.81, respectively. Minor Gb4Cer species endowed with C16:1, C18:0, C20:0, C22:1, C24:2, and C26:1/C26:0 acyl chains that correspond to *m*/*z* values at 1247.72, 1277.77, 1305.81, 1331.82, 1357.82, and 1387.88/1389.90, respectively, were identified all carrying sphingosine (d18:1) in their ceramide moieties (data not shown). In addition to the elucidated GSLs, a sphingomyelin (SM) species with Cer (d18:1, C16:0) was co-purified with the neutral GSLs by means of anion-exchange chromatography and detected in the MS^1^ spectrum within the mass range of the MHC (Figure 2). Examples of CID-verified exact structures of Stx-binding Gb3Cer and Gb4Cer species are provided showing the MS^2^ spectra of Gb3Cer (d18:1, C16:0) and Gb4Cer (d18:1, C22:0) in Figures S2 and S3, respectively, in the Supplementary Materials, accompanied by explanatory fragmentation schemes. The pentahexosylceramide, which was not recognized by the two Stx-subtypes, was identified as Gb5Cer (Galβ3GalNAcβ3Galα4Galβ4Glcβ1Cer). Alternative pentahexosylceramides, such as the Forssman GSL (GalNAcα3GalNAcβ3Galα4Galβ4Glcβ1Cer), could be unambiguously excluded based on the fragmentation pattern obtained by CID MS analysis. The CID spectrum of Gb5Cer (d18:1, C16:0) together with the corresponding fragmentation scheme is depicted as an example of the various Gb5Cer lipoforms in Figure S4 in the Supplementary Materials.

### 2.3. Lipid Composition of DRM and Non-DRM Fractions Obtained from pHRCEpiCs

**Figure 3:**
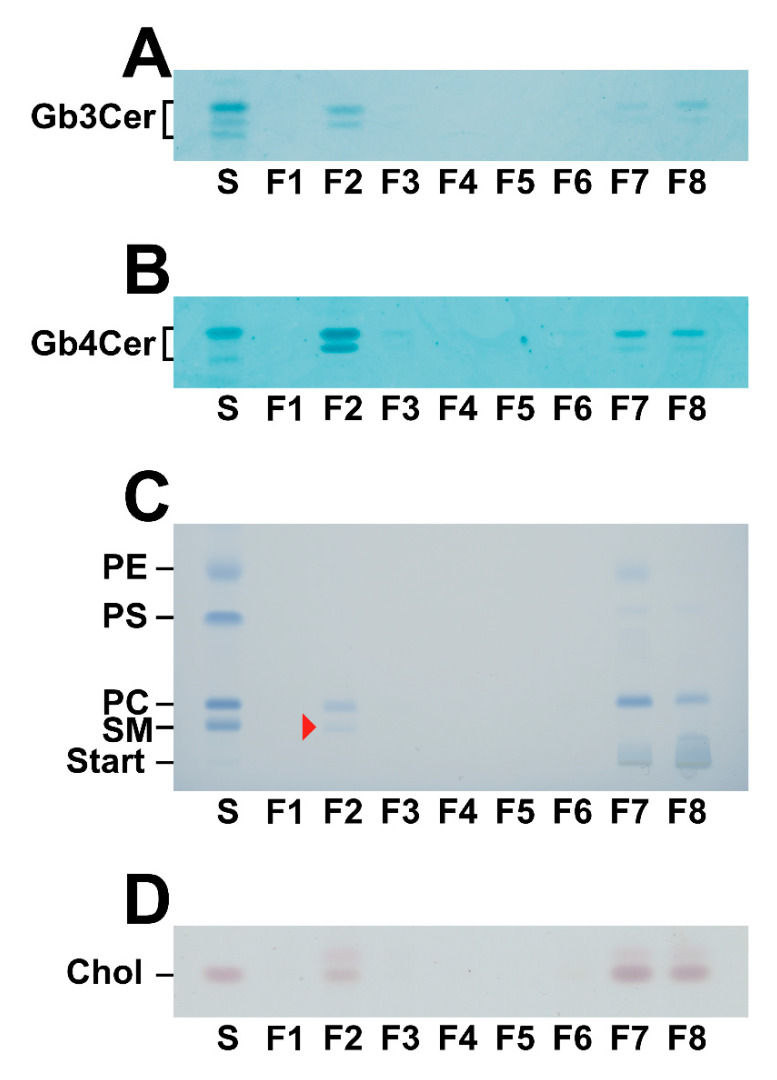
Distribution of Gb3Cer (**A**), Gb4Cer (**B**), phospholipids (**C**), and cholesterol (**D**) in sucrose gradient fractions F1 to F8 of pHRCEpiCs. Gradient fractions were prepared from replicate 1 of pHRCEpiCs (see R1 in Figure 1). GSL portions applied for the anti-Gb3Cer (**A**) and anti-Gb4Cer (**B**) TLC overlay assays corresponded to 4 × 10^5^ cells, respectively, and the amount of phospholipid staining (**C**) matched 2 × 10^6^ cells. The cholesterol staining (**D**) was equivalent to 1 × 10^6^ cells. The GSL standard mixture of neutral GSLs from human erythrocytes for the Gb3Cer (**A**) and Gb4Cer (**B**) immunochemical detection was equivalent to 2 and 0.2 µg, respectively. The phospholipid standard mixture is composed of phosphatidylethanolamine (PE), phosphatidylserine (PS), phosphatidylcholine (PC), and sphingomyelin (SM). The red arrowhead in F2 (**C**) marks SM and indicates its preference in classical detergent-resistant membrane (DRM) fraction F2. The phospholipid standard mixture corresponded to 20 µg and the cholesterol standard matched 0.5 µg of pure cholesterol. The phospholipids were stained with molybdenum blue and cholesterol with manganese (II) chloride. S, standard.

Possible association of Gb3Cer and Gb4Cer with membrane microdomains known as *lipid rafts* was probed by analyzing the distribution of the Stx receptors as well as ambient phospholipids and cholesterol to DRM and non-DRM fractions from sucrose gradient fractions of replicate 1 derived from pHRCEpiCs, as shown in Figure 3. The TLC overlay immunodetection of Gb3Cer and Gb4Cer in the GSL preparations of the eight gradient fractions F1 to F8 indicates prevalence of Gb3Cer and Gb4Cer in DRM fraction F2 (Figure 3A,B, respectively). Phosphatidylcholine (PC), the prominent phospholipid of pHRCEpiC membranes, was detected with the highest amount in the non-DRM fraction F7, and a lower content was found in the classical DRM fraction F2 (Figure 3C). The generally recognized *lipid raft* marker sphingomyelin (SM), although only weakly detectable and marked with an arrow head, was exclusively detectable in the DRM fraction F2, indicating a particular enrichment in the liquid-ordered phase of the membranes. A similar distribution like that of PC was found for cholesterol, which exhibited an obvious preference for the non-DRM fractions F7 and F8 accompanied by faint presence in F2 (Figure 3D). Thus, only the GSLs Gb3Cer and Gb4Cer as well as SM distributed to the classical DRM fraction F2, resembling the liquid-ordered membrane phase and suggesting their possible association with *lipid rafts*.

### 2.4. Mass Spectrometric Specification of the Stx Receptor GSLs Gb3Cer and Gb4Cer in DRM and Non-DRM Fractions Obtained from pHRCEpiCs

**Figure 4:**
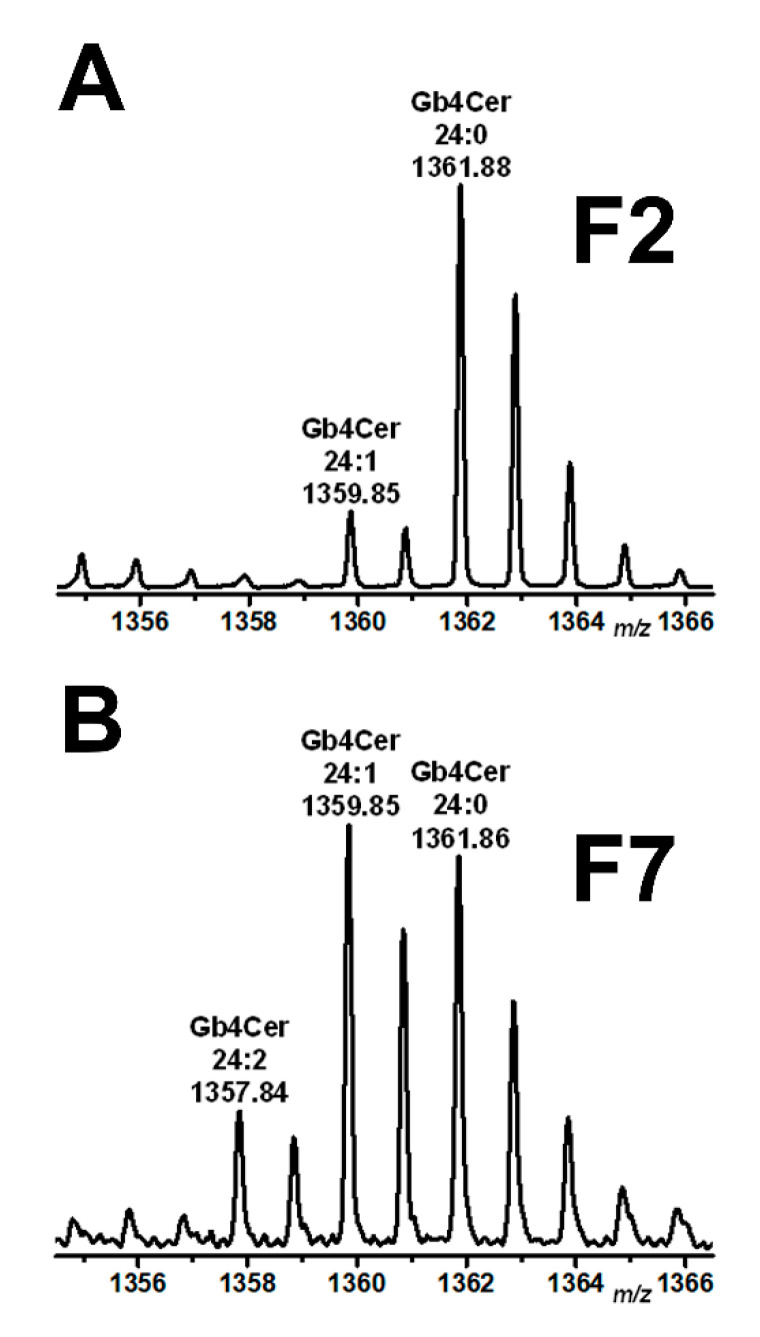
Partial MS^1^-spectra of Gb4Cer detected in DRM fraction F2 (**A**) and non-DRM fraction F7 (**B**) obtained from a sucrose gradient of pHRCEpiCs. Gradient fractions were prepared from replicate 1 of pHRCEpiCs (see Figure 3). The spectra were recorded in the positive ion mode and span the *m*/*z* range between 1356 and 1366 showing Gb4Cer lipoforms as singly charged monosodiated [M+Na]^+^ species with ceramide moieties built up from a constant sphingosine (d18:1) residue and varying C24 fatty acids as indicated.

In addition to the MS^1^ and MS^2^ analysis of the Stx-binding Gb3Cer and Gb4Cer species from total GSLs (see Figure 2), we performed MS^1^ and MS^2^ analysis of Gb3Cer and Gb4Cer from classical DRM fraction F2 and non-DRM fraction F7 of replicate 1 of pHRCEpiCs corresponding to the liquid-ordered and liquid-disordered membrane phase, respectively. The MS^1^ analysis of Gb3Cer and Gb4Cer of F2 revealed lipoforms carrying Cer (d18:1, C16:0) and Cer (d18:1, C22:0) as the less abundant and those harboring Cer (d18:1, C24:0) as the dominant GSL species accompanied by the minor variant with Cer (d18:1, C24:1) (not shown). The obtained pattern is comparable to that detected for Gb3Cer and Gb4Cer in the total GSL fraction (see Figure 2). Unfortunately, the F7 fraction was crowded by polyethylene glycols (PEGs) due to the usage of detergent that absolutely hampered the detection of Gb3Cer species, allowing only detection of low amounts of Gb4Cer endowed with Cer (d18:1, C16:0) and variations of the C24 fatty acid videlicet Cer (d18:1, C24:2), Cer (d18:1, C24:1), and Cer (d18:1, C24:0). Noteworthy is the observed shift in the degree of unsaturation of Gb4Cer with Cer (d18:1, C24:1/C24:0) from dominating C24:0 fatty acid in F2 to the prevalence of Gb4Cer with Cer (d18:1, C24:1/C24:0) with equal distribution of monounsaturated and saturated C24 fatty acid in F7, as shown in Figure 4A,B, respectively. Moreover, Gb4Cer (d18:1, C24:1/C24:0) was accompanied by the unusual Gb4Cer lipoform carrying doubly unsaturated C24:2 fatty acid (Figure 4B), which was detected neither in the total GSL fraction (see Figure 2) nor in the F2 fraction of pHRPTEpiCs (Figure 4A), underlining the preferred occurrence of Gb4Cer with a higher degree of unsaturation in the liquid-disordered membrane phase represented by the F7 fraction. The MS^2^ verification of the proposed structures is exemplarily shown for Gb4Cer (d18:1, C24:1/C24:0) of fraction F2 (see Figure 4A) in Figure S5 and for Gb4Cer (d18:1, C24:2/C24:1/C24:0) of fraction F7 (see Figure 4B) in Figure S6 of the Supplementary Materials, each accompanied by an explanatory fragmentation scheme.

### 2.5. Mass Spectrometric Specification of the Phospholipids and SM in DRM and Non-DRM Fractions Obtained from pHRCEpiCs

**Figure 5:**
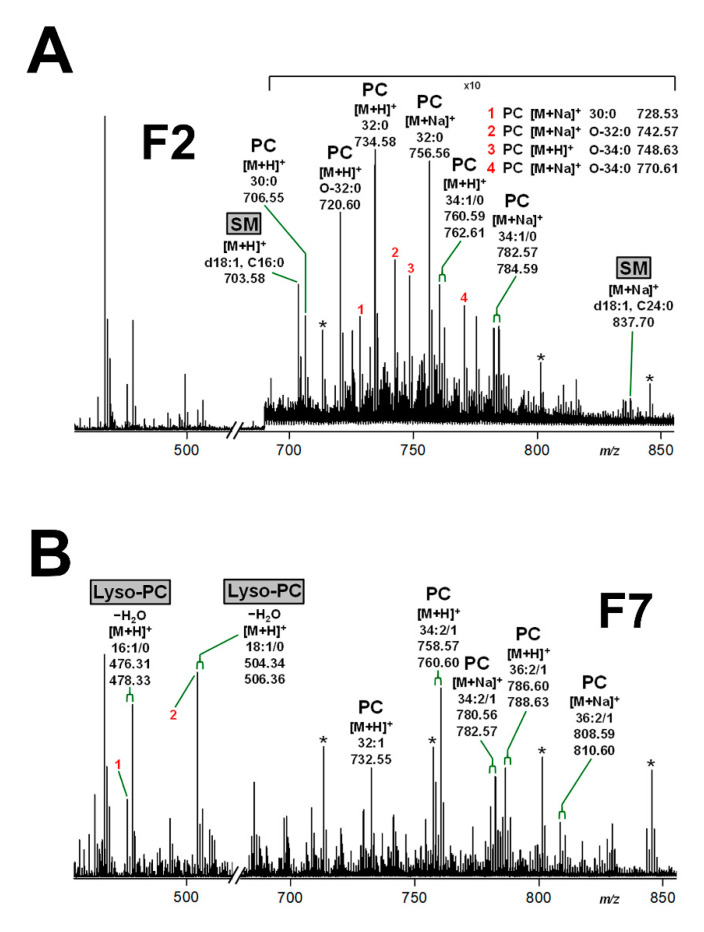
MS^1^-spectra of phospholipids detectable in DRM fraction F2 (**A**) and non-DRM fraction F7 (**B**) obtained from a sucrose gradient of pHRCEpiCs. Gradient fractions were prepared from replicate 1 of pHRCEpiCs (see Figure 3). The mass spectra were recorded in the positive ion mode, and phospholipids were detected as singly charged protonated [M+H]^+^ or monosodiated [M+Na]^+^ species as indicated in the spectra. The exclusive presence of SM and lyso-PC species, both highlighted as grayed boxes, in the DRM fraction F2 and the non-DRM fraction F7, respectively, indicates their specific distribution to the liquid-ordered and liquid-disordered membrane phase, respectively. Possible alternative structures for [M+Na]^+^ species at *m*/*z* 476.31 (**B**, 1) and 504.34 (**B**, 2) are lyso-PC (O-14:0) and lyso-PC (O-16:0), respectively. The asterisks indicate polyethylene glycols (PEGs).

The MS^1^ spectra of the lipid extracts of DRM fraction F2 and non-DRM fraction F7 obtained from pHRCEpiCs of replicate 1 (see Figure 3C) are shown in Figure 5. The MS^1^ spectrum of the detected phospholipids in fraction F2 (Figure 5A) shows protonated [M+H]^+^ and monosodiated [M+Na]^+^ species that could be assigned to PC lipoforms carrying different acyl and alkyl chains. More precisely, PC (32:0) accompanied by PC (O-32:0) with saturated acyl and alkyl chains showed highest abundance in the spectrum, accompanied by less abundant PC (30:0) and PC (O-34:0) as well as minor PC (34:1) with a monounsaturated acyl residue. This collection of phospholipids was flanked by ions that could be assigned to SM (d18:1, C16:0) and SM (d18:1, C24:0), highlighted by grayed boxes, whereof the latter appeared with minute signal intensities. The SM variants were detectable only in DRM fraction F2 (see also Figure 3C) representing DRM-specific lipids, which might be *lipid raft*-associated structures.

In the MS^1^ spectrum of the non-DRM bottom fraction F7 of pHRCEpiCs (Figure 5B), the [M+H]^+^ and [M+Na]^+^ species could be assigned to PC (34:2/1) with doubly and monounsaturated acyl chains, which dominated slightly over PC (36:2/1) and PC (32:1). Importantly, PC lipoforms with saturated acyl chains and SM were undetectable. Further highly abundant ions could be identified as lyso-PC (16:1/0) and lyso-PC (18:1/0), marked as grayed boxes, which are each characterized by the loss of an acyl chain. Alternative structures of the respective lyso-PC variants with monounsaturated 16:1 and 18:1 acyl chain are lyso-PC (O-14:0) and lyso-PC (O-16:0), respectively, each with a C2-shortened alkyl chain compared to the proposed species carrying an acyl chain. The lyso-PC species can be considered as reliable markers of the non-DRM fraction F7 and thus of the liquid-disordered membrane phase, since they were undetectable in the DRM fraction F2 (see Figure 5A).

### 2.6. Stx1a- and Stx2a-Mediated Cellular Damage of pHRCEpiCs

**Figure 6:**
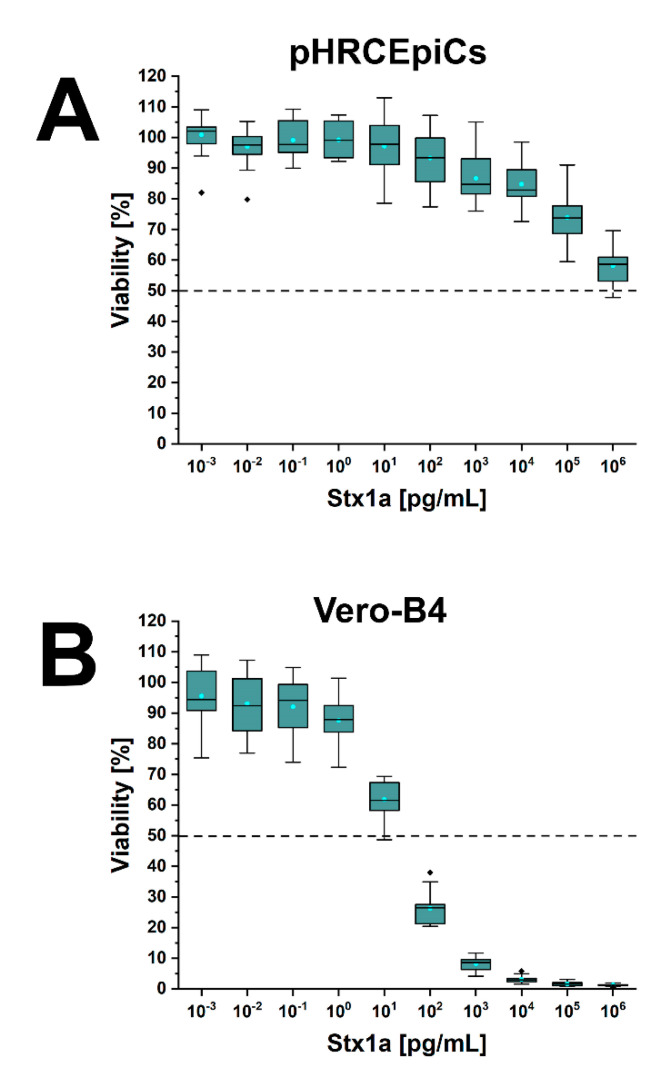
Cytotoxic action of Stx1a towards pHRCEpiCs (**A**) and Vero-B4 reference cells (**B**). Cytotoxicity was determined using the crystal violet assay, and absorption readings obtained from Stx1a-treated cells are displayed as a box plot diagram providing percentage values related to 100% viability of parallel cell cultures without toxin. Measurements of three biological replicates were performed as six-fold determinations. The 18 data points per toxin concentrations are portrayed as triangles, the medians as solid lines, and the means as dashed lines.

**Figure 7:**
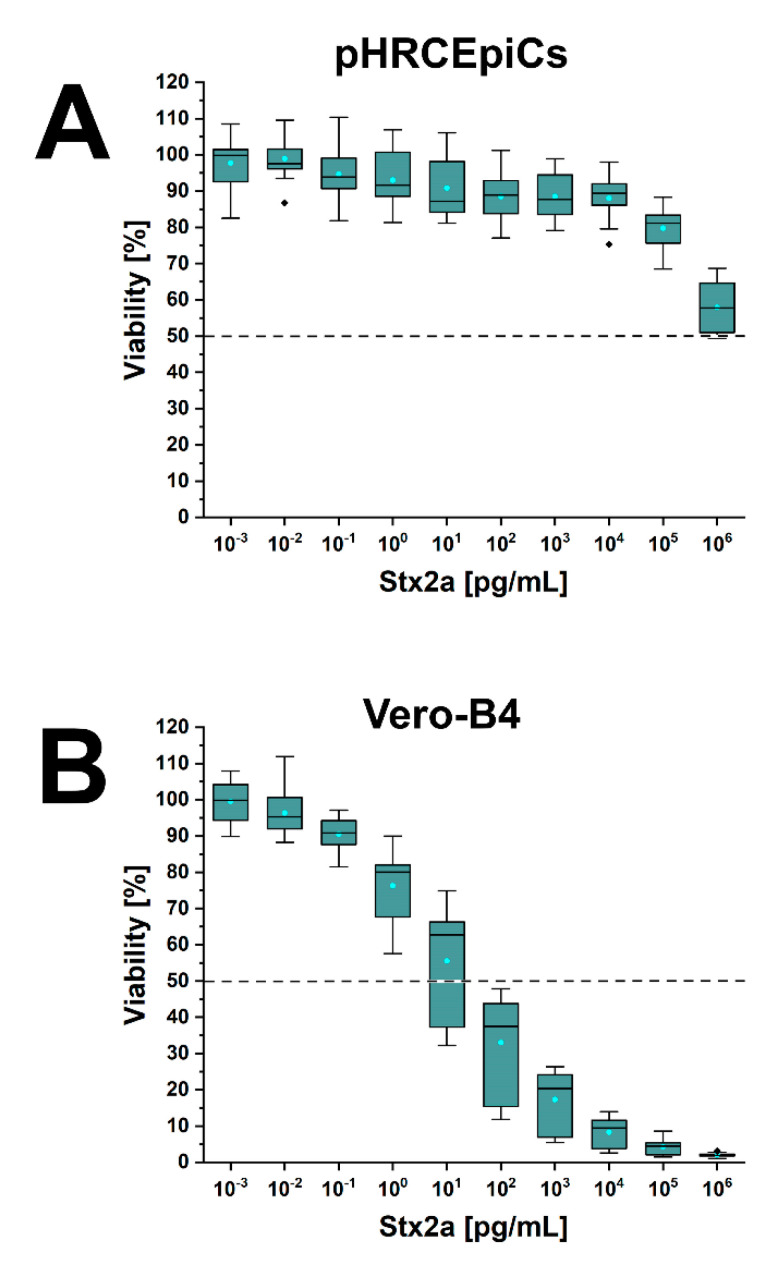
Cytotoxic action of Stx2a towards pHRCEpiCs (**A**) and Vero-B4 reference cells (**B**). Stx2a-mediated cytotoxicity was determined and plotted as outlined in the caption of Figure 6.

pHRCEpiCs were exposed to serial dilutions of decreasing concentrations of affinity-purified Stx1a and Stx2a ranging from 10^−3^ pg/mL (≡1 fg/mL) up to 10^6^ pg/mL (≡1 µg/mL). The cell viability of treated cells was determined in relation to cell cultures without toxin equivalent to 100% viability. The effects of Stx1a and Stx2a on the viability of pHRCEpiCs are portrayed in Figure 6 and Figure 7, respectively, and compared with those obtained in parallel cultures of Stx-treated Vero-B4 cells as positive controls. At low and moderate concentrations in the range from 10^−3^ to 10^1^ pg/mL of Stx1a, pHRCEpiCs were virtually resistant towards the toxin (Figure 6A). A first, weak response was detected upon exposure of the cells to 10^2^ pg/mL of Stx1a that gradually increased at higher toxin concentration, reaching a killing rate of 42 ± 6% (corresponding to a 58 ± 6% survival rate) at 1 µg/mL. The extrapolated 50% cytotoxic dose (CD_50_) for Stx1a was 1.51 µg/mL for Stx1a. By comparison, a clear and pronounced concentration-dependent reduction in cell viability was observed for Vero-B4 cells, starting at low toxin concentrations (1 pg/mL) and reaching a 38 ± 6% lethal rate (corresponding to 62 ± 6% cell survival) at 10 pg/mL (Figure 6B). The calculated CD_50_ was 13.3 pg/mL This indicates a much higher susceptibility of Vero-B4 cells towards Stx1a, amounting to approximately five orders of magnitude and reaching a de facto 100% killing rate with 0.1 and 1 µg/mL of Stx1a.

A significant Stx2a-induced decline in cell viability was observed at high Stx2a concentrations of 10^5^ and 10^6^ pg/mL (Figure 7A). Application of 1 µg/mL of Stx2a resulted in a final cell viability of 56 ± 15 % with an extrapolated CD_50_ of 1.36 µg/mL. This survival rate was observed for Vero-B4 cells at 10^1^ pg/mL (Figure 7B). The serial data curve was almost identical to that obtained with Stx1a (see Figure 6B) with a CD_50_ of 1.25 × 10^1^ pg/mL for Stx2a. This indicates an approximately five orders of magnitude higher sensitivity of Vero-B4 cells to Stx2a in comparison to pHRCEpiCs. Collectively, pHRCEpiCs were largely robust towards treatment with Stx1a and Stx2a at low and moderate toxin concentrations and showed a significant susceptibility merely at Stx1a concentrations ≥100 ng/mL.

## 3. Discussion

Although a number of studies dealing with Stx-mediated injury of human renal epithelial cells have been published, the structural details of the Stx-binding GSLs of human kidney epithelial cells have been left open for a long time, and the receptors of Stx were only superficially known. The various lipoforms of the Stx-receptor GSLs Gb3Cer and Gb4Cer of the human kidney epithelial cell lines A498, ACHN, and Caki-2 have been recently characterized by us [65,114], while the Stx-binding GSLs of primary human renal epithelial cells are hitherto unknown. We now fill this lack of knowledge with a comprehensive structural characterization of the Stx receptors Gb3Cer and Gb4Cer of pHRCEpiCs. In addition, we performed a fine analysis of the vicinal lipids that co-localize with Stx-binding Gb3Cer and Gb4Cer in the liquid-ordered and the liquid-disordered membrane phase of pHRCEpiCs represented by DRM and non-DRM fractions, respectively. This practice, i.e., the usage of detergent to isolate microdomains from cell membranes as DRMs and separating or differentiating them from non-DRMs, still represents a convenient methodological approach for assigning *lipid raft* affinity [86,88,89,111]. However, DRMs have merely an informative character on the possible clustering of GSLs, providing a convenient platform for molecular carbohydrate-binding processes such as GSL–toxin interaction. Noteworthy, DRMs do not necessarily reflect the real membrane composition of *lipid rafts*, and particular caution is recommended about claiming evidence for the GSL association with *lipid rafts* alone from their occurrence in DRMs. Seeking the occurrence of marker lipids specific to the different membrane phases, SM could be clearly recognized as a marker for the liquid-ordered membrane phase due to its preference in DRM fraction F2. Lyso-PC, the mono-acylated descendant of the double-tailed PC, was identified as a specific marker in the non-DRM fraction F7 corresponding to the liquid-disordered membrane phase. This opposed separation is not a unique feature of pHRCEpiCs and has been previously reported for primary human brain and kidney endothelial cells [115,116], human lymphoid and myeloid cells [117], and the Vero-B4 and MDCK II epithelial cell lines [118,119]. Working with DRMs and non-DRMs, a shift from dominance of the saturated PC (32:0) and PC (O-32:0) lipoforms in the DRM fraction F2 to prevalence of the double- and monounsaturated PC (34:2/1) and PC (36:2/1) variants in the non-DRM fraction F7 was observed for pHRCEpiCs. Notably, a similar difference in the distribution of PC with saturated and unsaturated acyl chains has been recently detected in primary human brain microvascular endothelial cells (pHBMECs) [120]. These findings suggest the association of the saturated PC lipoforms with the liquid-ordered membrane phase, while the unsaturated PC species may preferentially distribute to the liquid-disordered membrane phase. Furthermore, we could show co-localization of Gb3Cer and Gb4Cer with SM in the DRM fractions F1 to F3, suggesting their distribution together with SM to the liquid-ordered membrane phase of pHRCEpiCs. A similar trend was recognized for the different lipoforms of Gb3Cer and Gb4Cer occurring predominantly or even specifically in the DRM fraction F2 or non-DRM fraction F7, which are considered as representatives of the liquid-ordered and liquid-disordered membrane phase, respectively. In this study, we could show that Gb3Cer and Gb4Cer of pHRCEpiCs with saturated C16:0, C22:0, and C24:0 fatty acids dominated in DRM fraction F2, whereas lipoforms carrying unsaturated C24:1 and C24:2 fatty acids prevailed in the non-DRM fraction, as shown for Gb4Cer. The same specific distribution has been previously reported for primary endothelial cells of the human brain (pHBMECs) [120].

Common to all types of primary human endothelial and epithelial cells, including Vero-B4 kidney epithelial cells derived from African green monkeys [118], thus far analyzed by us is the preponderance of the Gb3Cer and Gb4Cer lipoforms with Cer (d18:1, C16:0) and Cer (d18:1, C24:1/C24:0), while species with fatty acyl chains harboring intermediate chain length from C18 to C22 were detected as less abundant compounds in the respective GSL preparations. The biological relevance and, in particular, the biological function of this stable feature of endothelial and epithelial cells remain largely unknown. Interestingly, the Gb3Cer and Gb4Cer lipoforms carrying the long-chain C24:1 or C24:0 fatty acid could be involved in a so-called interdigitation between the fatty acyl chains of the two leaflets of the cellular membrane. Recent results provided evidence that an interaction between long-chain (glyco)sphingolipids in the outer leaflet and phosphatidylserine (18:0/18:1) in the inner leaflet, a mechanism known as “hand-shaking” of the fatty acyl chains [121], may give rise to the formation of clusters by cross-linking sphingolipids that in turn can result in the transfer of signals to the cytosol [122]. It is therefore tempting to speculate about a possible specific role of Gb3Cer (d18:1; C24:1/C24:0) and Gb4Cer (d18:1, C24:1/C24:0) in Stx-mediated signal transduction, which remains to be uncovered, while the role of the shorter counterparts of Gb3Cer and Gb4Cer with Cer (d18:1/C16:0) remains enigmatic.

Stx-mediated damage of endothelial cells in the kidneys and the brain is central in the pathogenesis of HUS caused by EHEC infections [1,18,123,124]. More precisely, glomerular endothelial cells in the kidney and microvascular endothelial cells in the brain are preferably targeted by Stx leading to acute renal impairment and cerebral disturbances resulting in severe extraintestinal complications of STEC infections [12,13,38,45,50,125,126]. Because the renal epithelium may also be involved in the pathogenesis of Stx-mediated HUS, in vitro studies using primary kidney-derived epithelial cells have become increasingly recognized [13,46,127,128]. Besides the reported sensitivity of Stx to immortal epithelial cell lines of renal origin (not further discussed here), few studies have been performed so far, which provided essential knowledge on Stx-mediated damage of cultured primary human tubular [51,66,67,70,71,72,129,130,131] and glomerular epithelial cells [68,69]. Although being limited in their lifetime in vitro, primary cells offer few advantages when compared to immortal cell lines. Natural cells resemble much better the original tissue and correspond more closely to the in vivo situation. For this reason, primary human blood outgrowth endothelial cells and primary porcine brain endothelial cells are highly recommended in vitro models to study Stx-mediated damage close to the human in vivo situation [132] and to investigate the Stx-mediated collapse of the endothelial blood–brain barrier in pigs [133]. Considering the kidney epithelium, the development of 3D-cultures of primary human cortical renal tubular epithelial cells that resemble original human renal proximal tubules is a novel in vitro model to study renal epithelial repair mechanisms upon Stx-mediated injury [72]. In addition, ex vivo cultivated primary human hematopoietic stem/progenitor cells represent the ideal approach of unravelling the cytotoxic effects of Stxs towards the development of erythrocytes, a process known as erythropoiesis, which normally takes place in vivo in the bone marrow [39,40]. On the other hand, it should not be left unmentioned that primary cells are significantly more demanding than cell lines, as has been discussed in detail in a previous review by Legros and colleagues [12].

Regarding primary human renal epithelial cells, we cultivated pHRCEpiCs under serum-reduced conditions in medium supplemented with a cocktail of growth-promoting ingredients (but unknown exact composition due to trade secret of the provider) from passages 2 to 8, during which time they were phenotypically stable. Upon exposure to increasing concentrations of Stx1a and Stx2a (applied as serial dilutions of decreasing toxin concentrations) from 1 fg/mL up to 1 µg/mL, pHRCEpiCs were found largely robust against Stx-mediated cellular damage at low toxin concentrations in the range between 10^−3^ and 10^1^ pg/mL. First signs of responsiveness were recognized when applying Stx1a and Stx2a at ≥100 pg/mL and ≥100 ng/mL, respectively, which continuously increased to 42% cell killing (corresponding to 58% viability) at the highest toxin concentration of 1 µg/mL deployed in this study. The classical CD_50_, equal to 50% of cell survival, could therefore only extrapolated to theoretical values of 1.51 and 1.36 µg/mL for Stx1a and Stx2a, respectively. This rather low susceptibility was different to data from some previous publications but was also consistent with few studies of other groups working with natural kidney epithelial cells. Primary human proximal tubular epithelial cells were highly sensitive to the cytotoxic effect of Stx1 comparable to Vero cells and exhibited a CD_50_ of 100 pg/mL [129,130]. On the other hand concentrations of 100 ng/mL of Stx2 were required to cause an approximate 50% cell killing using human renal tubular epithelial cells [70]. In another study, primary pediatric renal tubular epithelial cells were found to respond on Stx2 challenge with evidence of apoptosis or necrosis at concentrations above 10 pg/mL, while 100 pg/mL of Stx2 exhibited marked cellular changes, such as membrane blebbing and disappearance of microvilli [51]. Human renal cortical epithelial cells in primary culture were found to be sensitive to Stx1 and Stx2 and developed signs of apoptosis at toxin concentrations of 100 pg/mL, respectively [66], or at even one order of magnitude lower concentrations when exposing primary human renal proximal tubular epithelial cells to Stx1 [67]. Probing the cytotoxic effects of Stx2 versus *E. coli* subtilase cytotoxin, the employed primary cultures of human cortical renal tubular epithelial cells were significantly more susceptible to the cytotoxic action of Stx2 than subtilase cytotoxin, reaching an approximate CD_50_ of 100 pg/mL for Stx2 [131]. A study dealing with the Stx1-mediated cellular damage of primary human glomerular epithelial cells revealed a slight decrease in cell viability when applying an Stx1 concentration of 100 ng/mL and a similar result for simultaneously performed cytotoxicity studies using primary human cortical tubular epithelial cells [68]. However, a CD_50_ of approximately 100 pg/mL has been reported in another study where primary human glomerular epithelial cells were exposed to Stx1 [69]. Despite the varying extent of cellular damage determined for Stx1(a) and Stx2(a) in primary cell cultures of different types of human kidney epithelial cells, the data indicate the possible involvement of Stx1(a) and/or Stx2(a) in tubular and glomerular damage in HUS. However, it seems that renal epithelial cells might not play the prime role in the manifestation of HUS.

## 4. Conclusions

The results indicate that presence of Stx-binding glycosphingolipids per se and prevalent occurrence in microdomains does not necessarily lead to a high cellular susceptibility towards Stx. Moreover, our results suggest that renal cortical epithelial cells might not play a major role in Stx-mediated kidney injury during the development of HUS. With our study, our goal was to add a further piece to completing the puzzle of the complex process of kidney failure in the development of HUS. However, data based on primary cells should not be generalized due to possible batch-to-batch heterogeneity of cell preparations derived from different donors. Furthermore, GSL expression can differ according to various culture conditions, suggesting that results from primary cells may not be necessarily representative for native tissues. Nevertheless, a systematic investigation on a large cohort of individual donors remains to be conducted. As a basic principle, primary kidney epithelial cells from different sources could serve for a systematic investigation on individually varying glycosylation profiles with focus on GSLs and differing responsiveness to Stx subtypes at least of clinically relevant Stx1a and Stx2a.

## 5. Materials and Methods

### 5.1. Cultivation and Propagation of pHRCEpiCs

Primary human renal cortical epithelial cells (pHRCEpiCs) were obtained from ScienCell^TM^ (Carlsbad, CA, USA; Cat. No. 4110). The lower case “p” signifies the status of “primary” cells to distinguish these kidney epithelial cells from tumor-derived or immortalized cell lines, such as A498, ACHN, or Caki-2 [65,114]. The pHRCEpiCs of our master bank, established from the 1st passage upon receipt and stored as cryopreserved aliquots in the gas phase over liquid nitrogen, were thawed and cultured in a humidified air atmosphere with 5% CO_2_ at 37 °C in ScienCell^TM^ epithelial cell medium (EpiCM, Cat. No. 4101) supplemented with 2% fetal bovine serum (FBS, Cat. No. 0010) and 1% epithelial cell growth supplement (EpiCGS, Cat. No. 4152) without antibiotics. The cells were passaged at approximate 80% confluence using 0.25% Trypsin-EDTA (Lonza, Verviers, Belgium; cat. CC-5012) according to standard procedures [134,135]. For the purpose to gain sufficing cell material for obtaining pure GSLs from lipid extracts of total cells and for the production of DRM and non-DRM fractions from sucrose density gradients (see below), two vials of pHRCEpiCs of the master bank, each equivalent to 5 × 10^5^ cells, were thawed, and cells were propagated in 175 cm^2^ tissue culture flasks (Greiner Bio-One, Frickenhausen, Germany) in EpiCM until passage 8. Microscopical control was performed with the aim of early recognition of senescence and dedifferentiation that might occur during prolonged cultivation of primary cells (see Figure S1 in the Supplementary Materials). Cells were continuously monitored using an Axiovert 40C microscope (Carl Zeiss AG, Oberkochen, Germany) and recorded with a digital camera (Canon PowerShot G10, Canon, Tokyo, Japan). Images were documented with AxioVison 4.8 (Zeiss) and processed with Adobe Photoshop software (Adobe Systems, San Jose, CA, USA). The Vero-B4 reference cell line, obtained from the German Collection of Microorganisms and Cell Cultures (DSMZ, Braunschweig, Germany; DSMZ no. ACC 33), was cultured in chemically defined serum-free OptiPRO^TM^ SFM medium (Gibco Life Technologies Corporation, Paisley, UK; catalogue no. 12309-019) supplemented with 4 mM L-glutamine and routinely passaged by trypsinization just before reaching confluence as described above.

### 5.2. Stx Cytotoxicity Assay

The crystal violet assay was applied for investigating Stx1a- and Stx2a-mediated cellular damage as previously described [44,118,134,136]. In short, trypsinized cells were dispersed to 96-well tissue culture plates (Corning Inc., Corning, NY, USA) in 100 µL volumes each corresponding to 4 × 10^3^ cells/well and allowed to settle for 24 h (37 °C, 5% CO_2_). Afterwards, the cells were exposed for 1 h to decreasing concentrations of affinity-purified Stx1a or Stx2a [136] in final volumes of 200 µL per cavity starting with the highest concentration of 1 µg/mL declining in 1:10 dilutions to a final toxin concentration of 1 fg/mL. Cell culture medium without toxin served as a control. After toxin treatment, the supernatant was replaced by fresh culture medium, and the cells were further incubated for 72 h. The cultivation was stopped by removal of the spent medium, and the remaining adhering cells were fixed with formalin. Staining with crystal violet and densitometrical quantification were performed as previously described in detail [44,118,134,136]. Results represent the means ± standard deviations (SD) of 6-fold determinations of 3 biological replicates and are displayed as percentage values in relation to untreated control cells set to 100% viability. The Stx concentration, which caused a damaging effect in 50% of the cells, was defined as the 50% cytotoxic dose (CD_50_).

### 5.3. Harvest of DRM and Non-DRM Fractions from Sucrose Density Gradients

The modus operandi of harvesting DRM and non-DRM fractions from sucrose density gradients obtained from pHRCEpiCs followed the classical procedure published by Brown and Rose [84] with minor modifications as previously described [115,117,118,120,135]. In brief, confluent grown cell monolayers were ruptured in lysis buffer, and the cell debris was eliminated by sparing centrifugation (400× *g*), followed by short-time ultracentrifugation (150,000× *g*) of the supernatant to separate the cellular membranes from the cytosol. After solubilization of the membrane sediment in 1% Triton X-100 buffer, the slurry was mixed with an identical volume of 85% sucrose. The resulting 42.5% sucrose solution was then consecutively overlayed with 30% and 5% sucrose followed by ultracentrifugation of the discontinuous sucrose gradient (200,000× g). Afterwards, three DRM top fractions (F1 to F3) and five lower non-DRM fractions (F4 to F8), each 1.5 mL in volume, were collected one after another top down from the gradient and submitted to lipid analysis (see below).

### 5.4. Isolation and Purification of GSLs from Total Cells

Lipids were extracted from two independently produced batches of pHRCEpiCs propagated in 175 cm^2^ cell culture flasks according to previously published protocols [44,114,115,118,135]. Concisely, the extraction started with methanol treatment of the confluent grown cell monolayers and was continued by successive extraction with chloroform/methanol mixtures of increasing chloroform content, videlicet chloroform/methanol (1/2, *v/*v**), chloroform/methanol (1/1, *v/*v**) and chloroform/methanol (2/1, *v/*v**). The pooled extracts were rotary evaporated, followed by disintegration of co-extracted alkali-labile triglycerides and phospholipids using 1 M methanolic NaOH for saponification (1 h, 37 °C). Afterwards, the sample was neutralized with 10 M HCl, dialyzed against deionized water, and freeze-dried. The dried desalted extract was resolved in chloroform/methanol/water (30/60/8, *v/*v*/*v**), and neutral GSLs were separated from acidic GSLs by means of anion-exchange chromatography using DEAE-Sepharose CL-6B (GE Healthcare, Munich, Germany) as described previously [137]. The preparation of neutral GSLs was taken up in chloroform/methanol (2/1, *v/*v**) and stored in a screw-capped vial with a Teflon seal at −20 °C until use.

### 5.5. Obtaining Phospholipid and GSL Preparations from DRM and Non-DRM Fractions

This procedure has been described in a number of previous publications [115,117,118,120,135] and is shortly explained. The gradient fractions F1 to F8 (see above) were dialyzed for 2 days at 4 °C against deionized water to remove the sucrose. Aliquots of 0.5 mL of each fraction were used for phospholipid analysis and submitted to lyophilization. The dried samples were then dissolved under sonication in chloroform/methanol (2/1, *v*/*v*) and adjusted to defined volumes corresponding to 1 × 10^5^ cells/µL. Further 0.5 mL-sized aliquots of the desalted gradient fractions, required for GSL and cholesterol analysis, were freeze-dried and incubated with agitation for 1 h at 37 °C in 1 N methanolic NaOH to saponify the alkali-sensitive phospholipids and triglycerides, followed by neutralization with 10 N HCl. The samples were then desalted by dialysis, freeze-dried, and taken up in chloroform/methanol (2/1, *v*/*v*) in a concentration equivalent to 1 × 10^5^ cells/µL.

### 5.6. Stx1a, Stx2a, Antibodies, and Lipid References

Stx1a and Stx2a (termed Stx1 and Stx2 in former publications, now changed to Stx1a and Stx2a according to the improved nomenclature of Scheutz and collaborators [113]) were affinity-purified from Stx-containing bacterial liquid culture supernatants of the *E. coli* wild-type strains of serotype O145:H-(strain 2074/97) and O111:H-(strain 03-06016), respectively [136]. The mode of action of the two affinity-purified Stx subtypes regarding receptor recognition and exertion of cytotoxic effects towards various cell types has been recently shown [12,65,120].

The polyclonal chicken IgY anti-Gb3Cer and anti-Gb4Cer antibodies employed for TLC overlay detection of globo-series GSLs have been described in numerous previous studies [24,65,119,138]. Anti-Stx1 and anti-Stx2 monoclonal mouse IgG antibodies (clone VT109/4-E9 and clone VT 135/6-B9, 2.75 mg/mL) were bought from SIFIN GmbH (Berlin, Germany). Affinity-purified polyclonal rabbit anti-chicken IgY (Code 303-055-033) and goat anti-mouse IgG (Code 115-055-003) alkaline phosphatase (AP)-labeled secondary antibodies were obtained from Dianova (Hamburg, Germany).

A sample of neutral GSLs from human erythrocytes served as positive control for the detection of Gb3Cer (Galα4Galβ4Glcβ1Cer) and Gb4Cer (GalNAcβ3Galα4Galβ4Glcβ1Cer) [117,139,140,141]. Cholesterol (Sigma Aldrich, Steinheim, Germany; cat. no. C8667) and a collection of defined phospholipids consisting of phosphatidylethanolamine (PE), phosphatidylserine (PS), phosphatidylcholine (PC), and sphingomyelin (SM) were used as references for lipid TLC analysis of the DRM and non-DRM fractions prepared from sucrose gradients following previous descriptions [117,118,119].

### 5.7. Thin-Layer Chromatography, Lipid Staining, and Overlay Detection

Isolated GSLs from total pHRCEpiCs as well as phospholipid and GSL preparations from DRM and non-DRM fractions were separated on glass-backed high-performance thin-layer chromatography (TLC) plates coated with silica gel 60 (HPTLC plates, size 10 cm × 10 cm, thickness 0.2 mm, no. 1.05633.0001; Merck, Darmstadt, Germany). Samples were spread to the silica gel layer using an automatic sample applicator (Linomat 5, CAMAG, Muttenz, Switzerland). The solvent chloroform/methanol/water (120/70/17, *v*/*v*/*v*) was employed for the separation of neutral GSLs, chloroform/methanol/isopropanol/triethylamine/0.25% aqueous KCl (30/9/25/18/6, each by vol.) for phospholipids and chloroform/acetone (96/4, *v*/*v*) for cholesterol. GSLs were stained with orcinol, phospholipids with molybdenum blue Dittmer–Lester reagent, and cholesterol with manganese(II)chloride after TLC separation, as outlined in previous papers [44,114,120].

The anti-Gb3Cer and anti-Gb4Cer TLC overlay assays were carried out with polyclonal chicken anti-Gb3Cer and anti-Gb4Cer antibodies as well as using Stx1a and Stx2a with the corresponding anti-Stx1 and anti-Stx2 antibody (see above), respectively, according to previously published protocols [39,118,142]. In short, after chromatography and silica gel fixation, the TLC plate was overlayed with 1:2000 diluted primary anti-GSL antibodies or solutions containing 0.33 µg/mL of affinity-purified Stx1a or Stx2a (see above). Bound primary anti-Gb3Cer and anti-Gb4Cer antibodies were detected with a secondary AP-conjugated anti-chicken IgY antibody (1:2000 dilution), and bound Stx1a and Stx2a were detected with 1:1000 diluted anti-Stx1 and anti-Stx2 antibody, respectively, followed by incubation with a secondary AP-conjugated goat anti-mouse IgG antibody (1:2000 dilution). Bound secondary antibodies were detected with 0.05% (*w/v*) 5-bromo-4-chloro-3-indolyl phosphate *p*-toluidine salt (BCIP, Roth, Karlsruhe, Germany) in glycine solution (pH 10.4), which generates a blue precipitate at sites of bound anti-GSL antibodies or Stxs on the TLC plate [44,65,120,143].

### 5.8. Mass Spectrometry of GSLs

A SYNAPT G2-S mass spectrometer (Waters, Manchester, UK) equipped with a Z-spray source was employed for nano-electrospray ionization mass spectrometry (nanoESI MS) of GSLs, as reported in detail in previous studies [24,25,65]. Briefly, dried aliquots of GSL preparations from total cells as well as DRM and non-DRM fractions were resolved in chloroform/methanol (1/4, *v*/*v*) and analyzed using the positive ion sensitivity mode. The source settings were as follows: temperature 80 °C, capillary voltage 0.8 kV, sampling cone voltage 20 V, and offset voltage 50 V. Low energy collision-induced dissociation (CID) MS^2^ experiments were performed aimed at the verification of GSL structures proposed from MS^1^ analysis. To this end, GSL precursor ions selected in the quadrupole analyzer were separated by ion mobility at the following parameters: wave velocity 700–800 m/sec, wave height 40 V, nitrogen gas flow rate 90 mL/min, and helium gas flow rate 180 mL/min. Subsequent fragmentation was performed in the transfer cell with collision energies of 70 to 100 eV (E_lab_). The nomenclature initiated by Domon and Costello was used for the assignment of the fragment ions obtained by MS^2^ analyses [144,145].

## Figures and Tables

**Table 1:** Major GSLs and SM of pHRCEpiCs determined by mass spectrometry combined with TLC immunodetection ^a^.

Compound ^b^	Ceramide	Formula	*m*/*z*_exp_^c^	*m*/*z*_calc_^c^
SM	d18:1, C16:0	C_39_H_79_N_2_O_6_PNa	725.56	725.5573
MHC	d18:1, C16:0	C_40_H_77_NO_8_Na	722.55	722.5547
MHC	d18:1, C20:0	C_44_H_85_NO_8_Na	778.61	778.6173
MHC	d18:1, C22:0	C_46_H_89_NO_8_Na	806.65	806.6486
MHC	d18:1, C24:1	C_48_H_91_NO_8_Na	832.67	832.6642
MHC	d18:1, C24:0	C_48_H_93_NO_8_Na	834.67	834.6799
Lc2Cer	d18:1, C16:0	C_46_H_87_NO_13_Na	884.60	884.6075
Lc2Cer	d18:1, C22:0	C_52_H_99_NO_13_Na	968.70	968.7014
Lc2Cer	d18:0, C24:1	C_54_H_101_NO_13_Na	994.72	994.7171
Lc2Cer	d18:0, C24:0	C_54_H_103_NO_13_Na	996.73	996.7327
Gb3Cer	d18:1, C16:0	C_52_H_97_NO_18_Na	1046.67	1046.6603
Gb3Cer	d18:1, C22:0	C_58_H_109_NO_18_Na	1130.76	1130.7542
Gb3Cer	d18:1, C24:1	C_60_H_111_NO_18_Na	1156.78	1156.7699
Gb3Cer	d18:1, C24:0	C_60_H_113_NO_18_Na	1158.78	1158.7855
Gb4Cer	d18:1, C16:0	C_60_H_110_N_2_O_23_Na	1249.74	1249.7397
Gb4Cer	d18:1, C22:0	C_66_H_122_N_2_O_23_Na	1333.83	1333.8336
Gb4Cer	d18:1, C24:1	C_68_H_124_N_2_O_23_Na	1359.85	1359.8493
Gb4Cer	d18:1, C24:0	C_68_H_126_N_2_O_23_Na	1361.87	1361.8649
Gb5Cer	d18:1, C16:0	C_66_H_120_N_2_O_28_Na	1411.79	1411.7925
Gb5Cer	d18:1, C22:0	C_72_H_132_N_2_O_28_Na	1495.89	1495.8864
Gb5Cer	d18:1, C24:1	C_74_H_134_N_2_O_28_Na	1521.91	1521.9021
Gb5Cer	d18:1, C24:0	C_74_H_136_N_2_O_28_Na	1523.92	1523.9177

^a^ The neutral GSL preparation was obtained from replicate 1 of pHRCEpiCs; TLC overlay detection of Gb3Cer and Gb4Cer was performed with Stx1a and Stx2a as well as anti-Gb3Cer and anit-Gb4Cer antibodies (see Figure 1); ^b^ all sphingolipids were detected in the positive ion mode as monosodiated [M+Na]^+^ species; exemplary MS^2^ spectra of Gb3Cer (d18:1, C16:0), Gb4Cer (d18:1, C22:0), and proposed Gb5Cer (d18:1, C16:0) are provided in Figure S2, Figure S3, and Figure S4, respectively, of the Supplementary Materials; ^c^ exp, experimental; calc, calculated.

## Supplementary Materials

The following are available online at https://www.mdpi.com/2072-6651/13/2/139/s1, Figure S1: Light microscopy micrographs of pHRCEpiCs during passage 3 (P3) and passage 15 (P15) at approximate 50% confluence, Figure S2: MS^2^ spectrum of Gb3Cer (d18:1, C16:0) (A) and corresponding fragmentation scheme (B) obtained from the neutral GSL preparation of pHRCEpiCs, Figure S3: MS^2^ spectrum of Gb4Cer (d18:1, C22:0) (A) and corresponding fragmentation scheme (B) obtained from the neutral GSL preparation of pHRCEpiCs, Figure S4: MS^2^ spectrum of Gb5Cer (d18:1, C16:0) (A) and corresponding fragmentation scheme (B) obtained from the neutral GSL preparation of pHRCEpiCs, Figure S5: MS^2^ spectrum of Gb4Cer (d18:1, C24:1/C24:0) (A) and corresponding fragmentation scheme (B) obtained from the F2 gradient fraction of pHRCEpiCs, Figure S6: MS^2^ spectrum of Gb4Cer (d18:1, C24:2/C24:1/C24:0) (A) and corresponding fragmentation scheme (B) obtained from the F7 gradient fraction of pHRCEpiCs.
