# Supplementary material for: Shiga Toxin (Stx)-Binding Glycosphingolipids of Primary Human Renal Cortical Epithelial Cells (pHRCEpiCs) and Stx-Mediated Cytotoxicity
Source: Toxins (Basel). 2021 Feb 12;13(2):139. doi: 10.3390/toxins13020139 (PMC7918848; doi:10.3390/toxins13020139)
Supplement: Supplementary file 1 [file toxins-13-00139-s001.pdf]

Supplementary Materials: Shiga Toxin (Stx)-Binding Glycosphingolipids of Primary Human Renal Cortical Epithelial Cells (pHRCEpiCs) and Stx-Mediated Cytotoxicity

Johanna Detzner, Elisabeth Krojnewski, Gottfried Pohlentz, Daniel Steil, Hans-Ulrich Humpf, Alexander Mellmann, Helge Karch and Johannes Müthing

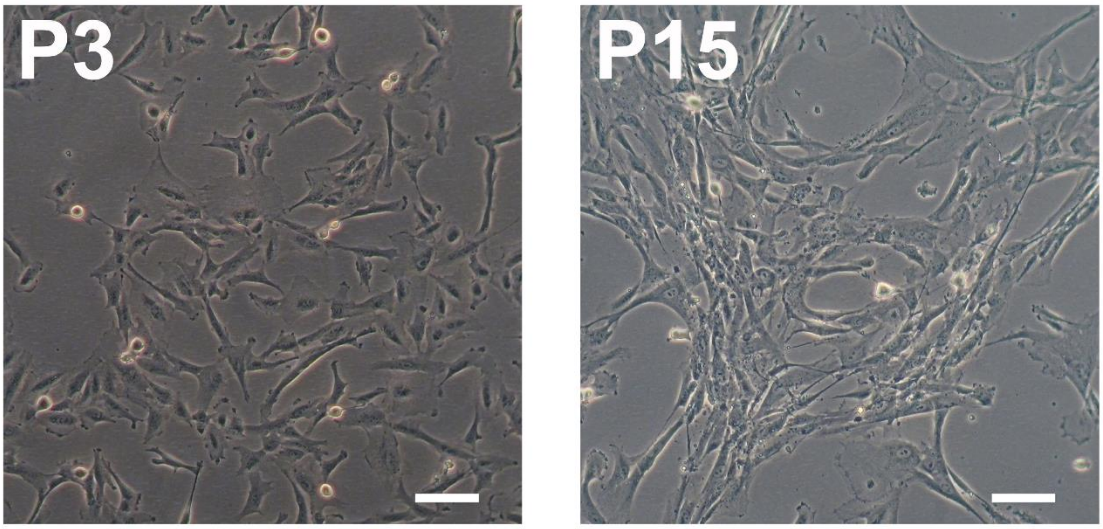

Figure S1. Light microscopy micrographs of pHRCEpiCs during passage 3 (P3) and passage 15 (P15) at approximate 50% confluence. Original magnification ×10. Bar: 100 µm.

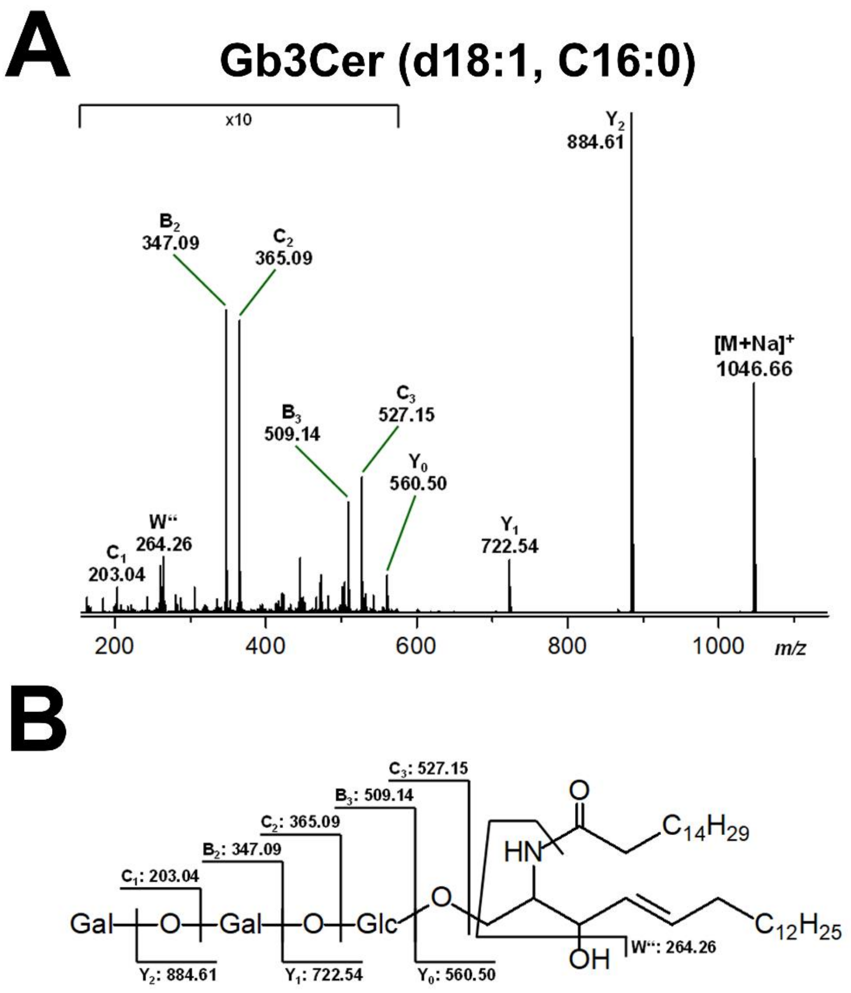

Figure S2. MS2 spectrum of Gb3Cer (d18:1, C16:0) (**A**) and corresponding fragmentation scheme (**B**) obtained from the neutral GSL preparation of pHRCEpiCs. The MS2 spectrum was achieved by CID experiments of the $[M+Na]^+$ precursor ions at *m*/*z* 1046.66 (see Figure 2) and illustrates, along with the explanatory fragmentation scheme, the structural proof of the MS1-based postulated Gb3Cer lipoform.

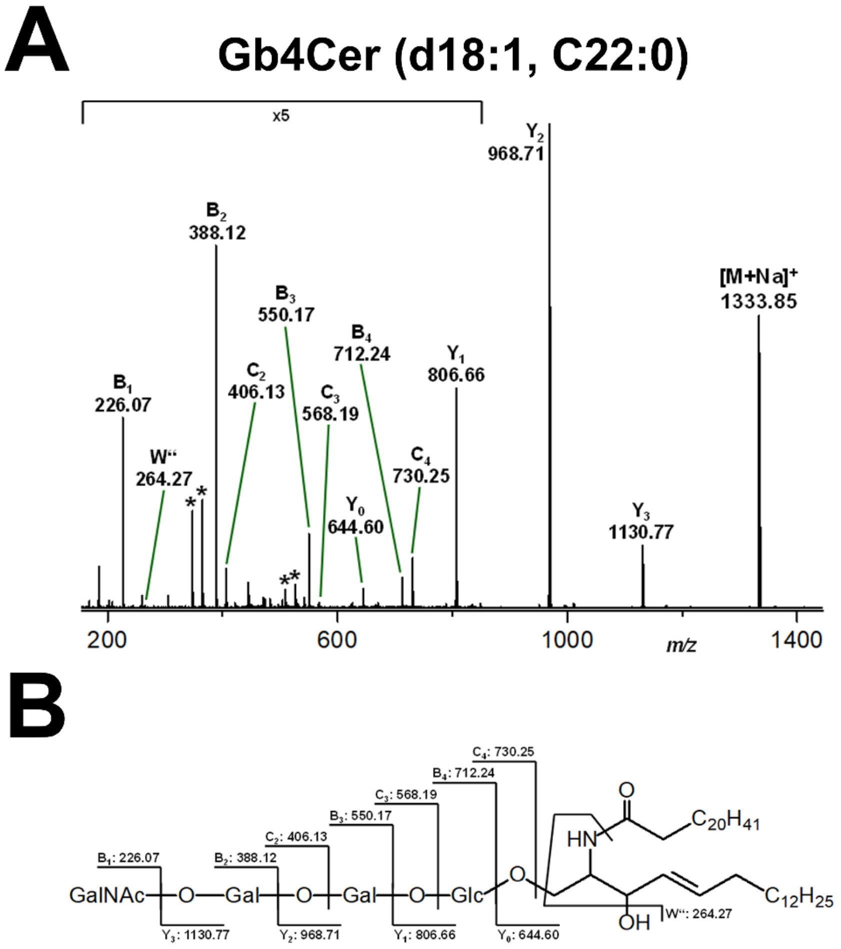

Figure S3. MS2 spectrum of Gb4Cer (d18:1, C22:0) (**A**) and corresponding fragmentation scheme (**B**) obtained from the neutral GSL preparation of pHRCEpiCs. The MS2 spectrum was achieved by CID experiments of the $[M+Na]^+$ precursor ions at *m*/*z* 1333.85 (see Figure 2) and illustrates, along with the explanatory fragmentation scheme, the structural proof of the MS1-based postulated Gb4Cer lipoform.

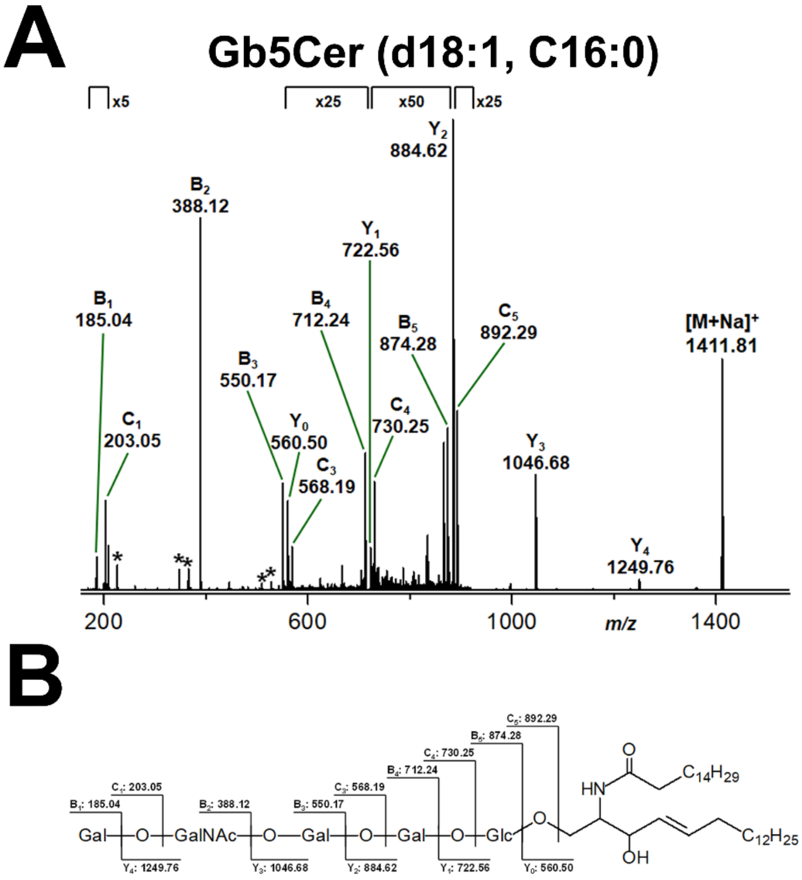

Figure S4. MS2 spectrum of Gb5Cer (d18:1, C16:0) (**A**) and corresponding fragmentation scheme (**B**) obtained from the neutral GSL preparation of pHRCEpiCs. The MS2 spectrum was achieved by CID experiments of the $[M+Na]^+$ precursor ions at *m*/*z* 1411.81 (see Figure 2) and illustrates, along with the explanatory fragmentation scheme, the structural proof of the MS1-based postulated Gb5Cer lipoform.

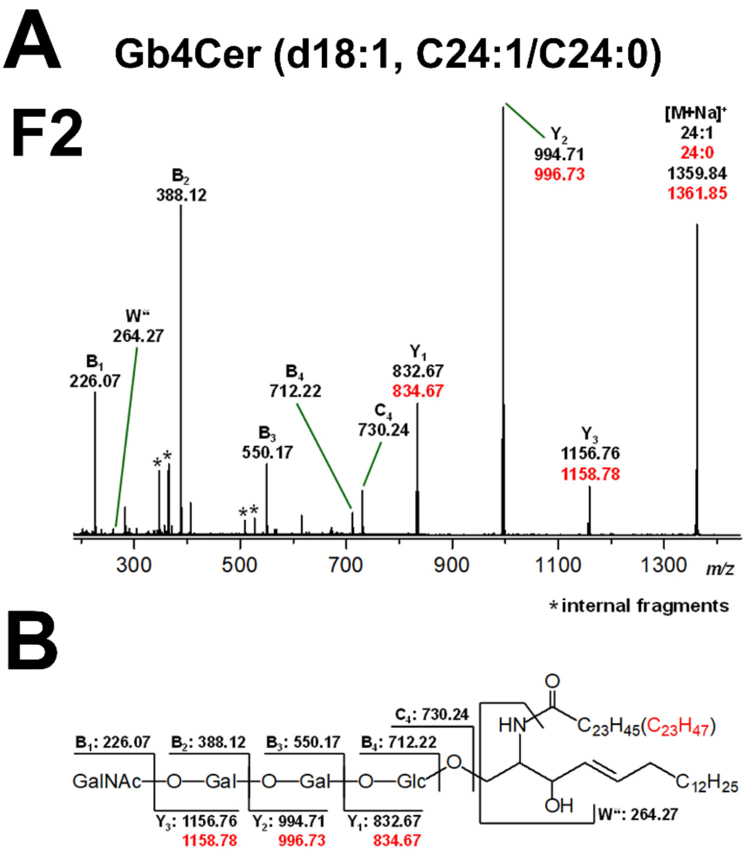

Figure S5. MS2 spectrum of Gb4Cer (d18:1, C24:1/C24:0) (**A**) and corresponding fragmentation scheme (**B**) obtained from the F2 gradient fraction of pHRCEpiCs. The MS2 spectrum was achieved by CID experiments of the $[M+Na]^+$ precursor ions at *m*/*z* 1359.84/1361.85 (see Figure 4) and illustrates, along with the explanatory fragmentation scheme, the structural proof of the MS1-based postulated Gb4Cer lipoforms.

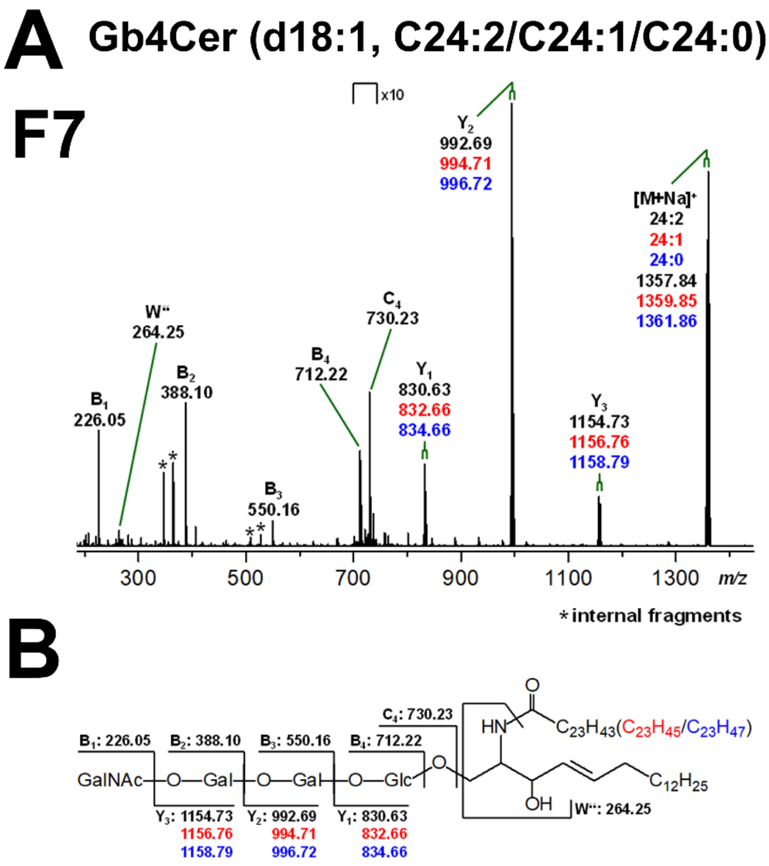

Figure S6. MS2 spectrum of Gb4Cer (d18:1, C24:2/C24:1/C24:0) (**A**) and corresponding fragmentation scheme (**B**) obtained from the F7 gradient fraction of pHRCEpiCs. The MS2 spectrum was achieved by CID experiments of the $[M+Na]^+$ precursor ions at *m*/*z* 1357.84/1359.85/1361.86 (see Figure 4) and illustrates, along with the explanatory fragmentation scheme, the structural proof of the MS1-based postulated Gb4Cer lipoforms.
